# Ozone alleviates MSU-induced acute gout pain via upregulating AMPK/GAS6/MerTK/SOCS3 signaling pathway

**DOI:** 10.1186/s12967-023-04769-1

**Published:** 2023-12-08

**Authors:** Wen Fan, Chong Liu, Dacai Chen, Chenjie Xu, Xiuting Qi, Ailin Zhang, Xuexian Zhu, Yujie Liu, Lei Wang, Lanxiang Hao, Wen-Tao Liu, Liang Hu

**Affiliations:** 1 Department of Pharmacology, School of Basic Medical Sciences, Nanjing Medical University, Nanjing, 211166 China; 2 Department of Anesthesiology and Pain, Nanjing First Hospital, Nanjing Medical University, Nanjing, 210006 Jiangsu China; 3 The Fourth Affiliated Hospital of Nantong University, Jiangsu, China; 4 Yancheng Ruikang Hospital, Jiangsu, 224000 China; 5 The Yancheng Clinical College of Xuzhou Medical University, The First People’s Hospital of Yancheng, Jiangsu, 224005 China; 6 Nanjing University of Chinese Medicine Institute of Literature in Chinese Medicine, Jiangsu, 224000 China

**Keywords:** Ozone, AMPK, Gas 6, MerTK, SOCS3, MMP9

## Abstract

**Background:**

Gout pain seriously affects the quality of patients' life. There is still no effective treatment. The inflammatory response is the main mechanism of gout. Here, we found that ozone can reduce the inflammatory reaction in the joints of gouty mice and relieve gout pain, and we further explore its protective mechanism.

**Methods:**

MSU was used to establish the gouty mice model. Nociception was assessed by Von Frey hairs. Cell signaling assays were performed by western blotting and immunohistochemistry. The mouse leukemia cells of monocyte macrophage line RAW264.7 were cultured to investigate the effects of ozone administration on macrophage.

**Results:**

Ozone reduced inflammation, relieved gout pain and improved the paw mean intensity and duty cycle of the gouty mice. Ozone increased the phosphorylation of AMP-activated protein kinase (AMPK), induced suppressor of cytokine signaling 3 (SOCS3) expression and inhibited metallopeptidase 9 (MMP9) expression. In vivo, ozone activated AMPK to induce Gas6 release, and upregulated MerTK/SOCS3 signaling pathway to reduce inflammation in mouse macrophage line RAW264.7. Inhibitors of AMPK and MerTK, respectively abolished the analgesic and anti-inflammatory effects of ozone in vivo and in vitro. Gas6 knockout cancelled the protectively effects of ozone on gout pain and the paw mean intensity and duty cycle of gouty mice. Additionally, the level of Gas6 and protein S in plasma of patients with hyperuricemia was significantly higher than that of healthy contrast group.

**Conclusion:**

Ozone reduces inflammation and alleviates gout pain by activating AMPK to up-regulate Gas6/MerTK/SOCS3 signaling pathway.

**Supplementary Information:**

The online version contains supplementary material available at 10.1186/s12967-023-04769-1.

## Introduction

Gout is characterized by hyperuricemia and recurrent inflammatory episodes caused by intra-articular crystal deposition of monosodium urate (MSU) [1]. The prevalence of gout has steadily increased due to risk factors such as diet, obesity, and an aging population, affecting ~ 6.8% of the global population [2]. Currently, steroidal and non-steroidal anti-inflammatory drugs, colchicine, and biologic control gout attacks. However, these drugs lack safety and have disadvantages like serious side effects, high costs, or poor analgesic effects on patients with comorbidities. Therefore, there is still a need for new medications for the treatment of gout.

Ozone therapy has been used in clinical practice to treat a variety of conditions, especially chronic pain [3]. Our previous studies have shown that medical ozone can reduce neuropathic pain [4] and treat chemoenteritis [5] by activating AMP-activated protein kinase (AMPK). PRKAG2, which encodes the γ^2^ chain of AMPK, shows a genomic hypomethylation pattern in peripheral blood mononuclear cells from patients with gouty arthritis [6]. Genetic AMPK α1 deficiency significantly increased the inflammatory response to MSU in mice in vivo. In addition, AMPK mediated colchicine anti-inflammatory effects in vitro [7]. These encourage us to position ozone as a candidate for therapeutic testing in the context of acute gouty arthritis (AGA).

Chronic low-grade inflammation is the key factor driving the pathogenesis of acute symptoms of AGA. Suppressor of cytokine signaling 3 (SOCS3)3 is an important participant in bone-related inflammation. Some studies have shown that the expression of SOCS3 in synovium tissue increases in Gout patients in the regression phase [8]. Animal-level studies demonstrated that mice with SOCS3 deficiency in the hematopoietic compartment showed the characteristics of inflammatory arthritis deterioration and cartilage damage [9]. Neutrophils and macrophages have the largest number in gout joint fluid and are the main effector cells that activate the innate immunity of the body [10]. They can react to the deposited MSU crystals through TLR2 and TLR4 [11]. A large number of studies have shown that SOCS3 protein is an effective inhibitor of TLR-driven immune response in macrophages [12]. We have previously demonstrated that AMPK activation can inhibit neuroinflammation by upregulating SOCS3 expression. Therefore, we speculate that ozone may improve experimental gout arthritis by activating AMPK and upregulating SOCS3. Considering that TAM receptors(Tyro3, Axl, and MerTK) are important upstream of SOCS3 that [13], on one hand, mediate the phagocytosis and clearance of macrophages to apoptotic cells, and on the other hand, inhibit SOCS3-activated inflammation, we set out to determine whether the upregulation of AMPK/SOCS3 by ozone is related to TAM.

Here we explored the molecular mechanism of ozone in treating gout pain by activating the TAM/SOCS3 signaling pathway. We found that ozone promoted the release of GAS6 from macrophages and activated the MerTK receptor, further inducing SOCS3 expression to treat gout pain. Here, we provided evidence for the treatment of experimental gout with ozone for the first time and a theoretical basis for the clinical application of ozone.

## Methods

### Ethics statement

All procedures were strictly performed in accordance with the regulations of the ethics committee of the International Association for the Study of Pain and the Guide for the Care and Use of Laboratory Animals (The Ministry of Science and Technology of China, 2006). All animal experiments were approved by Nanjing Medical University Animal Care and Use Committee and were approved by the Ethics Committee of Nanjing Medical University (No. IACUC-1908026). The study was approved by the institutional review board of Nanjing first hospital registered under KY20171228-KS-01 and all methods were carried out in accordance with relevant guidelines and regulations.

### Gouty arthritis induced by MSU crystal in mice

Adult male SPF ICR mice (20–22 g), 6–8 weeks old, were provided by the Experimental Animal Center of Nanjing Medical University. The mice were raised at 24 ± 2 °C, 55 ± 10% relative humidity, 12 h of light/12 h of dark circulation, and freely fed commercial standard food and tap water. All animal experiments have been approved by the Animal Protection and Use Committee of Nanjing Medical University, and strictly abide by the regulations of the Ethics Committee of the International Association for Pain Research and the guidelines for the care and use of experimental animals. MSU suspension (0.5 mg/10 μl) prepared by intra-articular injection (i.a) after each animal was anesthetized with 2.5% isoflurane to the right ankle joint to induce acute gout. The control group was injected with normal saline into the ankle joint. 20 min after MSU injection, each mouse in MSU experimental group was treated with ozone (i.p) of different doses.

### Behavioral studies

Ankle edema was assessed by an increase in ankle circumference, measured with a micrometer thickness gauge caliper (Mitutoyo). The result is expressed as Δmm, by calculating the difference between the test value and the basic value observed at different time points after the establishment of the gout model. Mechanical hyperalgesia was assessed using a series of Von Frey filaments (Aesthesio) and expressed as a mechanical withdrawal threshold (g).

The gait data were collected through the Gait Analysis and Processing System of ZhongShi Technology (ZS-BT/S) to evaluate the gait changes of mice. When collecting data, we ensured that the environment was kept dark enough for the machine to operate, with no interfering factors. After completing the measurement, the WalkAnalyzer was used to filter the required video clips, remove the impurities in the image that could affect the results, and correct the wrong footprints caused by system recognition. The video, walking sequence, timeline view and other required data were exported for analysis.

### Preparation and use of MSU crystal

We weighed 0.25 g sodium urate and 0.4 g NaOH, added them into 10 ml dd water and heated them to 70 ℃ to dissolve. After the temperature was naturally reduced to room temperature, we added hydrochloric acid to adjust the pH to 11.45. The supernatant was centrifuged (1500 r/min, 5 min) and collected immediately when the solution turned milky white with flocculent precipitation, then kept at 4 ℃ overnight. Precipitated crystals were then centrifuged and collected to be washed twice with 100% ethanol and placed in the drying oven for drying. The needle shape and size of the crystals were examined by a polarizing microscope. We re-suspended the MSU crystals in the culture medium and mixed them with ultrasound for cell experiments, and re-suspended them in normal saline and uniformly mixed them with ultrasound for animal experiments.

### Collection and processing of blood and ankle joint samples

After 6 h of ozone treatment, the mice were anesthetized with 4% pentobarbital and sacrificed for blood and tissue sample collection. The ankle joint was homogenized at 4 ℃ in RIPA buffer and was centrifuged at 14,000 rpm at 4° C for 15 min and the supernatant was stored at – 80 °C until further analysis. Ankle joint samples were fixed in 10% neutral buffered formalin for 48 h and then decalcified in EDTA decalcifying solution (Servicebio, Cat: G1105) for 3 weeks. The decalcifying solution was replaced every three days for staining.

### Preparation of medical ozone

Hermann medical ozone instrument was used to connect the oxygen tank to prepare medical ozone of different concentrations (mixture of ozone and oxygen). The volume of ozone injected intraperitoneally in each mouse was 0.5 ml.

### Chemicals and reagents

Antibody for Protein S and AMPK were from Santa Cruz Biotechnology (Santa Cruz, CA, USA)、Antibody for Gas6 (ABclonal), Antibody for SOCS3was from Abcam(Cambridge, MA, USA)、Antibody for pAMPK (Thr172) and pMerTK (Tyr749) were from Cell Signaling Technology(Beverly, MA, USA), Antibody for β-actin and Compound C (CC) was purchased from Sigma-Aldrich (St. Louis, MO, USA), UNC2541 and TP0903 were from MedChemExpress (Shanghai, China). Secondary antibodies were from Jackson ImmunoResearch Laboratories (West Grove, PA, USA). Fetal bovine serum (FBS) was purchased from Gibco, and other cell culture media and supplements were purchased from KenGEN (KenGEN BioTECH, China). All other reagents were from Sigma-Aldrich (St. Louis, MO, USA).

### Quantitative PCR

Quantitative PCR was performed on Raw264.7 cell and ankle joint samples obtained from mice. Total RNA was isolated using the standard method with TRIZOL reagent (Invitrogen Life Technologies). The isolated RNA was reverse-transcribed into cDNA using the PrimeScript™ RT Reagent Kit (TaKaRa) following standard protocols. Real-time quantitative PCR (qPCR) was performed using synthetic primers and SYBR Green (TaKaRa) on a QuantStudio 5 Real-Time PCR Detection System (Thermo Fisher Scientific). The relative expression levels of mRNA were calculated and quantified using the 2 − ΔΔCT method after normalization with the reference β-actin. The following primers were used:

- GAPDH: Forward:5′-GGCATGGACTGTGGTCATGAG-3′
- Reverse: 5′-TGCACCACCAACTGCTTAGC-3′
- IL-1β: Forward: 5′-TCATTGTGGCTGTGGAGAAG-3′
- Reverse: 5′-AGGCCACAGGTATTTTGTCG-3′
- TNF-α: Forward: 5′-CATCTTCTCAAAATTCGAGTGACAA-3′
- Reverse: 5′-TGGGAGTAGACAAGGTACAACCC-3′
- IL-6: Forward: 5′-ACTTCCATCCAGTTGCCTTCTTGG-3′
- Reverse: 5′-TTAAGCCTCCGACTTGTGAAGTGG-3′

### Data processing and analysis

All data were expressed in mean ± S.E.M. GraphPad Prism 9 software was used to perform one-way and two-way ANOVA on all data and combined with a t-test (Student Newman Keuls Test). Results were represented as mean ± SEM of three independent experiments. P < 0.05 was deemed to be statistically significant.

## Results

### Ozone alleviates gouty arthritis induced by MSU in mice

**Fig. 1:**
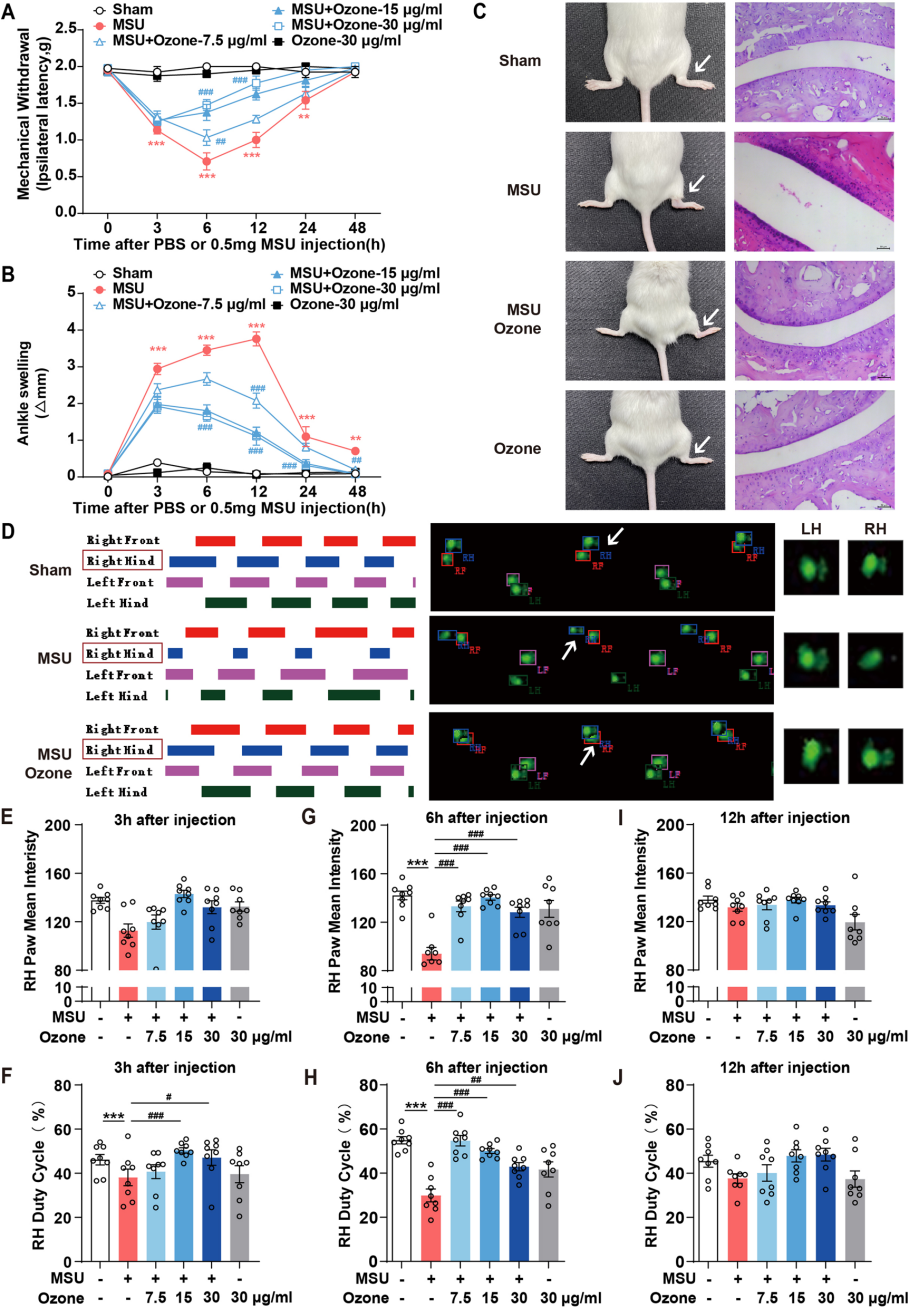
Ozone suppressed MSU-induced gout pain in mice. Mice (n = 8) were treated with various doses of ozone (i.p) 10 min after the injection of MSU crystals (0.5 mg/10 μl). **A** Mechanical allodynia was performed to evaluate the effect of ozone. **B** Time course of changes in MSU-induced ankle swelling. **C** Representative photographs and hematoxylin- and eosin-stained sections of the ankle joints obtained 6 h after MSU injection. **D** Representative footprint images were recorded during the treatment of Ozone. The colored bands represent standing time of each foot and the right panels are footprints of the right hind paw reflected by green LED light. **E**, **G**, **I** The paw mean intensity was analyzed by CatWalk software. RH, right hind. **F**, **H**, **J** The duty cycle was analyzed by CatWalk software. RH, right hind. ^*^p < 0.05, ^**^p < 0.01, ^***^p < 0.001 vs. sham group; ^#^p < 0.05, ^##^p < 0.01, ^###^p < 0.001 vs. the MSU-treated group

The animals in our study received MSU (0.5 mg/10 μl) Injections in the right ankle joints only. Consistent with our previous research [14], the injection of MSU crystals into the ankle joint will produce an acute inflammatory response, which can simulate human acute gouty arthritis. We used the traditional test method (von Frey) and function test (CatWalk) to evaluate the gout pain response induced by MSU and the therapeutic effect of ozone while observing the changes in ankle inflammation by evaluating the ankle circumference. The mechanical abnormal pain reaction caused by injection reached a peak at 6 h after injection and then subsided at 48 h (Fig. 1A). In contrast, there were no significant changes in the control group with vehicle-injected mice (Fig. 1B). In addition to the perception of injury, MSU (i.a) caused a threefold increase in the development of joint edema. As shown in Fig. 1B, the swelling of ankle joints reached a maximum of 12 h after the MSU injection. Compared with the control group, the ankle joint was obviously swollen at least until the time point of the final study, that is, 48 h. At 6 h after injection of MSU, compared with the Control group, MSU caused obvious redness and edema, which was relieved after ozone treatment (Fig. 1C).

To evaluate the role of ozone in MSU-induced inflammation in vivo, we used HE staining to investigate the inflammatory infiltration of mouse joints. The ankle joint of mice in the control group is intact, without obvious inflammatory cell infiltration (Fig. 1C). On the contrary, MSU significantly increased the infiltration of inflammatory cells to the ankle joint, while the MSU group was also accompanied by the widening of the cavity gap. Ozone treatment reduces the infiltration of inflammatory cells and joint edema. These results indicated that ozone can effectively improve the AGA model's pathological state of the ankle joint.

As compensation, patients tend to reduce the weight bearing of the affected limb when pain is caused by a certain stage of pain, which results in a shortened supporting period. In CatWalk, we observed that after MSU modeling, the paw mean intensity and duty cycle of the right foot print of mice 's right foot print decreased significantly (Fig. 1D), which was consistent with the previous report [15]. This reduction represents that the contact time between the right claw and the ground becomes shorter and the weight is significantly transferred from the arthritic part to the opposite limb, that is, the weight bearing is insufficient, which is consistent with the performance of pain avoiding gait. Similar to the animals in this study, the pain-induced inflammation of human feet will lead to the disappearance of heel contact [16]. However, after ozone treatment, the paw mean intensity and duty cycle were restored to normal (Fig. 1E–H). Pain, swelling, and improvement of gait behavior were observed when treated with any dose of ozone, but a dose of 15 μg/ml showed the best therapeutic effect at 6 h after modeling (Fig. 1G, H). These results indicate that ozone has a sufficient therapeutic effect on MSU-induced arthritis and support the use of 15 μg/ml doses in subsequent experiments.

### Ozone activates the AMPK/SOCS3 signal pathway and inhibits MMP9 expression to relieve gout pain

**Fig. 2:**
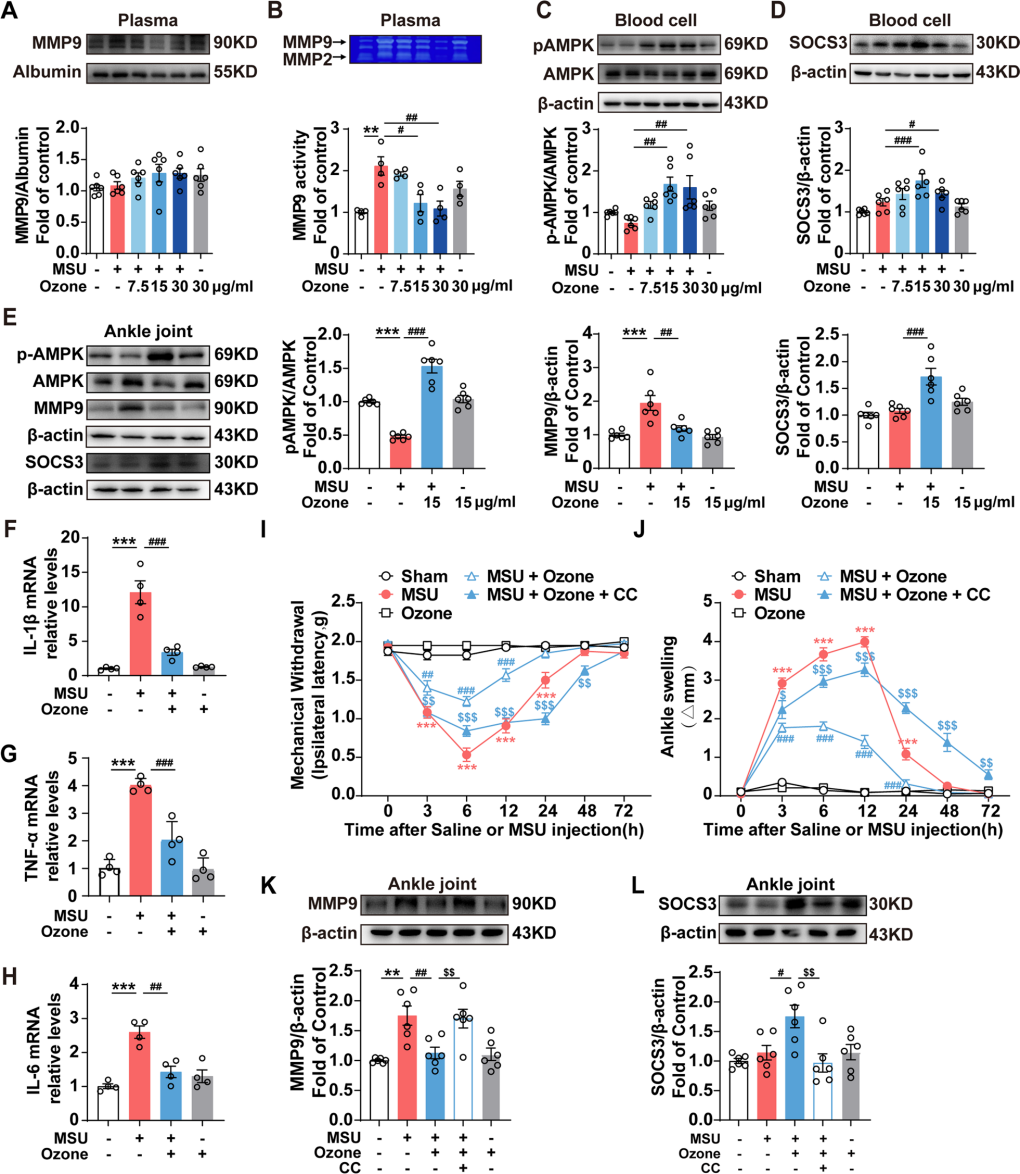
Ozone activated the AMPK/SOCS3 signal pathway and inhibited MMP9 expression to relieve gout pain. **A** After 6 h of indicated treatments, the MMP9 of mice 's plasma was tested using western blot. Albumin was used as a loading control. **B** MMP9 were measured with zymography assays. Blood cells (**C**, **D**) and ankle joints (**E**)with the indicated treatments were subjected to western blot assay of pAMPK, AMPK, SOCS3, MMP9 and β-actin. **F**–**H** Up/down-regulation of IL-1β, TNF-α and IL-6 mRNA were compared with the control group and MSU-treated group (n = 4). **I** Effect of ozone on MSU-induced mechanical withdrawal threshold combined with CC-treatment in GA mice. CC (i.p, 5 mg/kg) were primed 12 h before MSU injection. **J** Joint swelling gain at different time points. **K**, **L** Western blot analysis and the expression of MMP9 and SOCS3 in ankle joint. Representative western blot bands and a data summary are shown. ^*^p < 0.05, ^**^p < 0.01 and ^***^p < 0.001 vs. control group; ^#^p < 0.05, ^##^p < 0.01 and ^###^p < 0.001 vs. MSU-treated group; ^&^p < 0.05, ^&&^p < 0.01 and ^&&&^p < 0.001 vs. MSU + ozone group

We first wanted to explore the changes of AMPK in the blood cell of mice. In arthritic joints, matrix metalloproteinases (MMPs) and their inhibitors have been proven to play a role in the degradation of extracellular matrix (ECM) and tissue repair. More importantly, MMPs can promote IL-1 β, the Maturity of Caspase 3. MMP9 is an important marker for pain, which is both required and sufficient for mechanical pain symptoms17. In the mouse AGA model, although MMP9 showed no significant difference in the Western blot analysis of plasma (Fig. 2A), the gelatinase spectrum analysis showed that its activity was significantly increased and could be reversed by ozone (Fig. 2B). After the intraperitoneal injection of ozone, the blood AMPK increased significantly (Fig. 2C). Our previous results showed that the activation of AMPK could inhibit neuroinflammation by upregulating the expression of SOCS3 [4]. When we evaluated the expression of SOCS3, we found that SOCS3 in the ozone treatment group increased significantly (Fig. 2D). The results of blood also further supported that the best dosage of ozone was 15 μg/ml.

The expression level of AMPK protein in the ankle joint was significantly decreased in response to the stimulation of MSU crystal. On the contrary, MMP9 was significantly increased. Ozone could reverse these changes, which was related to the significant improvement of joint swelling and pain in the model of gouty arthritis induced by MSU (Fig. 2E). Quantitative PCR results suggest that, compared to the control, IL-1β, TNF-α and IL-6 were significantly high-regulated following treatment with MSU, and decreased when treated with ozone (Fig. 2F–H). In order to clarify are all these cytokines upregulated by the same transcription factor, we used PDTC (100 μM), an inhibitor of NF-κB. Quantitative PCR results showed that inhibition of NF-κB activation effectively suppressed MSU-mediated up-regulation of cytokines, including IL-1β, IL-6 and TNF-α, which simulated the therapeutic effects of ozone (Figure s1A-C). ELISA results showed that, compared to the control, IL-1β, TNF-α and IL-6 were high-secreted when treated with MSU, and decreased following treatment with ozone (Additional file 1: Fig. S1D–E).

We carried out further studies to confirm the contribution of AMPK/SOCS3 in alleviating pain caused by gout. Compared with the ozone treatment group, the overall reduction of mechanical withdrawal threshold (Fig. 2I) and the aggravation of swelling (Fig. 2J) were observed in the AMPK inhibition group. Further Western blot analysis showed that the decrease of MMP9 (Fig. 2K) and the increase of SOCS3 (Fig. 2L) in the ankle joint after ozone treatment were both canceled by CC, indicating that the inhibition of ozone-mediated inflammation depended on the activation of AMPK.

### Ozone activates AMPK/Gas6/MerTK/SOCS3 signaling pathway to inhibit MMP9 activity and inflammatory response in RAW264.7 cells

**Fig. 3:**
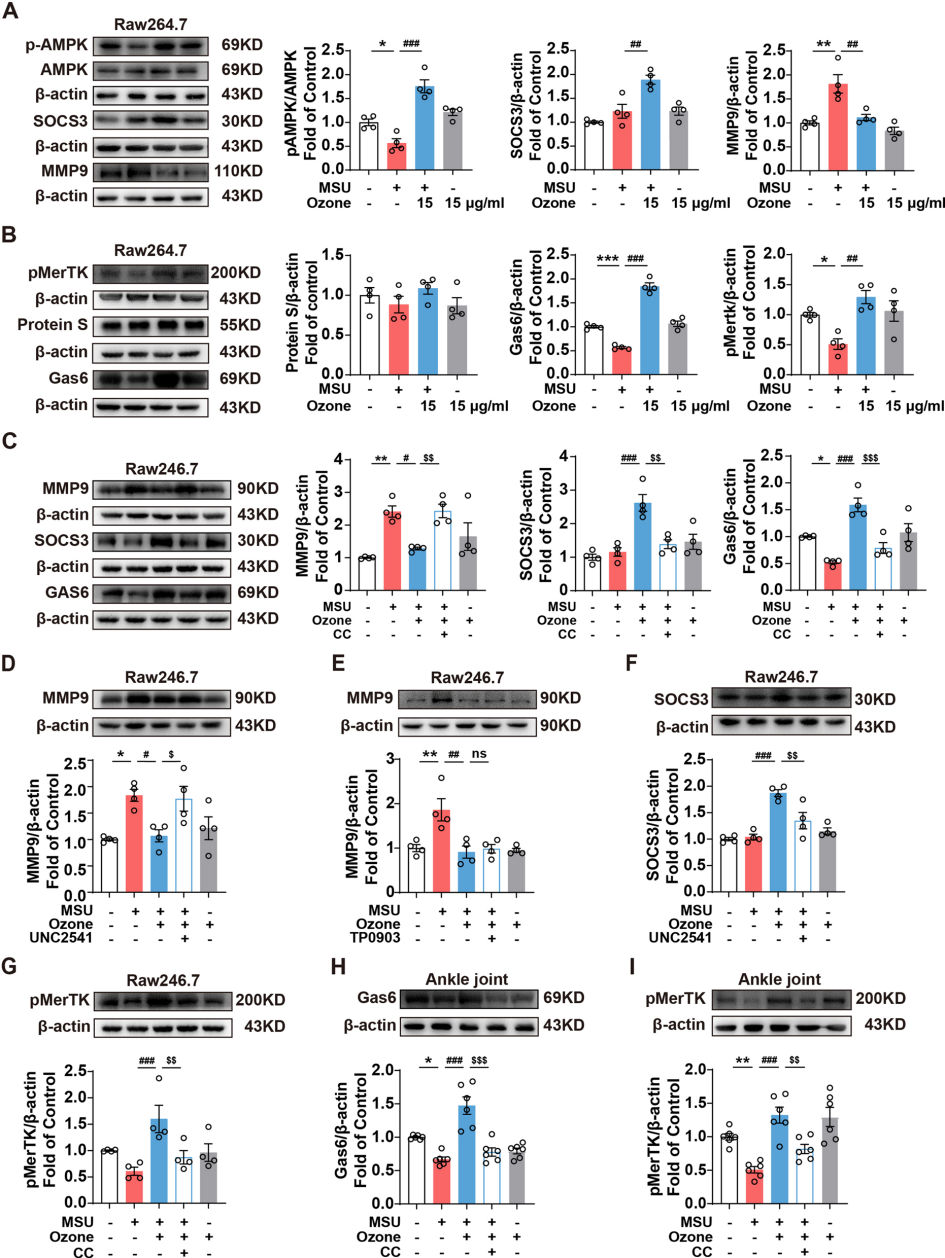
Ozone activated AMPK/Gas6/MerTK/SOCS3 signaling pathway to inhibit MMP9 activity and inflammatory response in RAW264.7 cells. Raw264.7 were primed with LPS (1 μg/ml, 6 h), and challenged with MSU suspension (200 μg/ml, 6 h) followed by treatment with CC (20 μM)/UNC2541(2.5 μM)/TP0903(100 nM). Ozone is given ten minutes after MSU treatment. **A** Western blot analysis and the expression of pAMPK, AMPK, SOCS3, MMP9 and β-actin. **B** Western blot analysis and the expression of pMerTK, Protein S, Gas6 and β-actin. **C** Western blot analysis showed that CC suppressed the expression of SOCS3, and Gas6 and Increase the expression of MMP9 in Raw264.7. **D**–**F** Expression of MMP9 and SOCS3 after UNC2541 or TP0903 treated in Raw264.7. **G** Phosphorylation of MerTK upon CC exposure in Raw264.7. **H**–**I** Expression of Gas6 and pMerTK after CC treated in the ankle joint. ^*^p < 0.05, ^**^p < 0.01 and ^***^p < 0.001 vs. control group; ^#^p < 0.05, ^##^p < 0.01 and ^###^p < 0.001 vs. MSU-treated group; ^&^p < 0.05, ^&&^p < 0.01 and ^&&&^p < 0.001 vs. MSU + ozone group

Consistent with the results of the ankle joint, compared with the MSU stimulation group, ozone can significantly induce SOCS3 expression and reduce MMP9 protein level and inflammatory factor expression level (Fig. 3A). We further explored the regulatory mechanism of ozone on inflammation.

Some studies have shown that the activation of the MerTK receptor can induce the expression of SOCS3 to play an anti-inflammatory role. We detected the expression level of MerTK in Raw264.7 after ozone treatment, the phosphorylation level of MerTK in Raw cells was significantly reduced after MSU modeling, and pMerTK was significantly activated after ozone treatment. The activation of TAM receptor depends on its binding to ligands Gas6 and Protein S. Our results showed that ozone can only promote the release of Gas6, not Protein S (Fig. 3B). Using the MerTK inhibitor UNC2541 (rather than Axl inhibitor TP0903), it was found that inhibition of MerTK can cancel the effect of ozone on SOCS3 and MMP9 (Fig. 3D–F). These data suggested that ozone up-regulates SOCS3 depending on Gas6/MerTK-mediated signaling.

We further explore the importance of AMPK in the ozone anti-inflammatory process and give AMPK inhibitor treatment before ozone treatment. The results are shown in the figure. Inhibiting AMPK can cancel the regulatory effect of ozone on GAS6, MerTK, SOCS3, and MMP9 (Fig. 3C). In addition, we detected the expression level of GAS6 and MerTK at the ankle joint of mice. Compared with the gout group, ozone can significantly increase the expression of GAS6 and activate the MerTK receptor. This effect was cancelled by AMPK inhibitor CC Fig. 3H, I), suggesting that ozone inhibited MMP9 activity and inflammatory reaction by activating AMPK to promote the release of Gas6 and up-regulate the MerTK/SOCS3 signal pathway.

### Ozone inhibits MMP9 activity through the MerTK/SOCS3 signal pathway to relieve gout

**Fig. 4:**
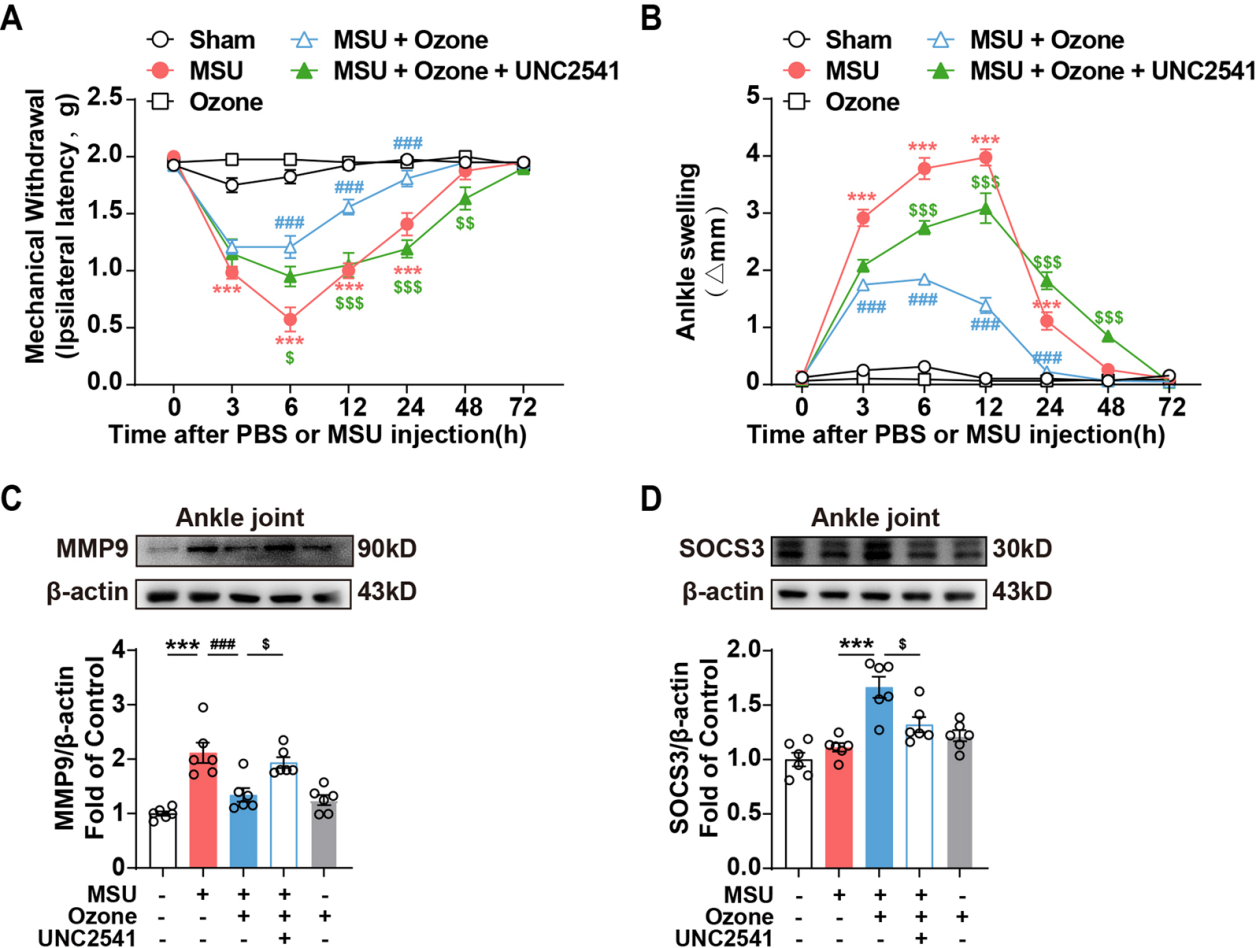
Ozone inhibited MMP9 activity through the MerTK/SOCS3 signal pathway to relieve gout. **A** Mechanical allodynia was performed to evaluate the effect of ozone after UNC2541 was treated. **B** Time course of changes in MSU-induced ankle swelling after UNC2541 was treated. **C**, **D** Western blot analysis showed the expression levels of MMP9 and SOCS3 after being treated with UNC2541 in the ankle joint. ^*^p < 0.05, ^**^p < 0.01 and ^***^p < 0.001 vs. control group; ^#^p < 0.05, ^##^p < 0.01 and ^###^p < 0.001 vs. MSU-treated group; ^&^p < 0.05, ^&&^p < 0.01 and ^&&&^p < 0.001 vs. MSU + ozone group

To evaluate the participation of the MerTK signaling pathway in AGA inflammation, we used the small molecule inhibitor UNC2541. In the model induced by MSU in vivo, inhibition of MerTK partially canceled the ozone-mediated analgesic and anti-inflammatory effects. It is mainly reflected in the prolonged detumescence (Fig. 4B) and recovery of mechanical hyperalgesia after the administration of inhibitors, as well as the lower pain threshold compared with the treatment group (Fig. 4A). The WB results of the ankle joint showed that it could partly reverse the inhibition of ozone on MMP9 (Fig. 4C) while cancelling the rise of SOCS3 (Fig. 4D).

### Gas6 plays a key role in relieving gout pain with ozone

**Fig. 5:**
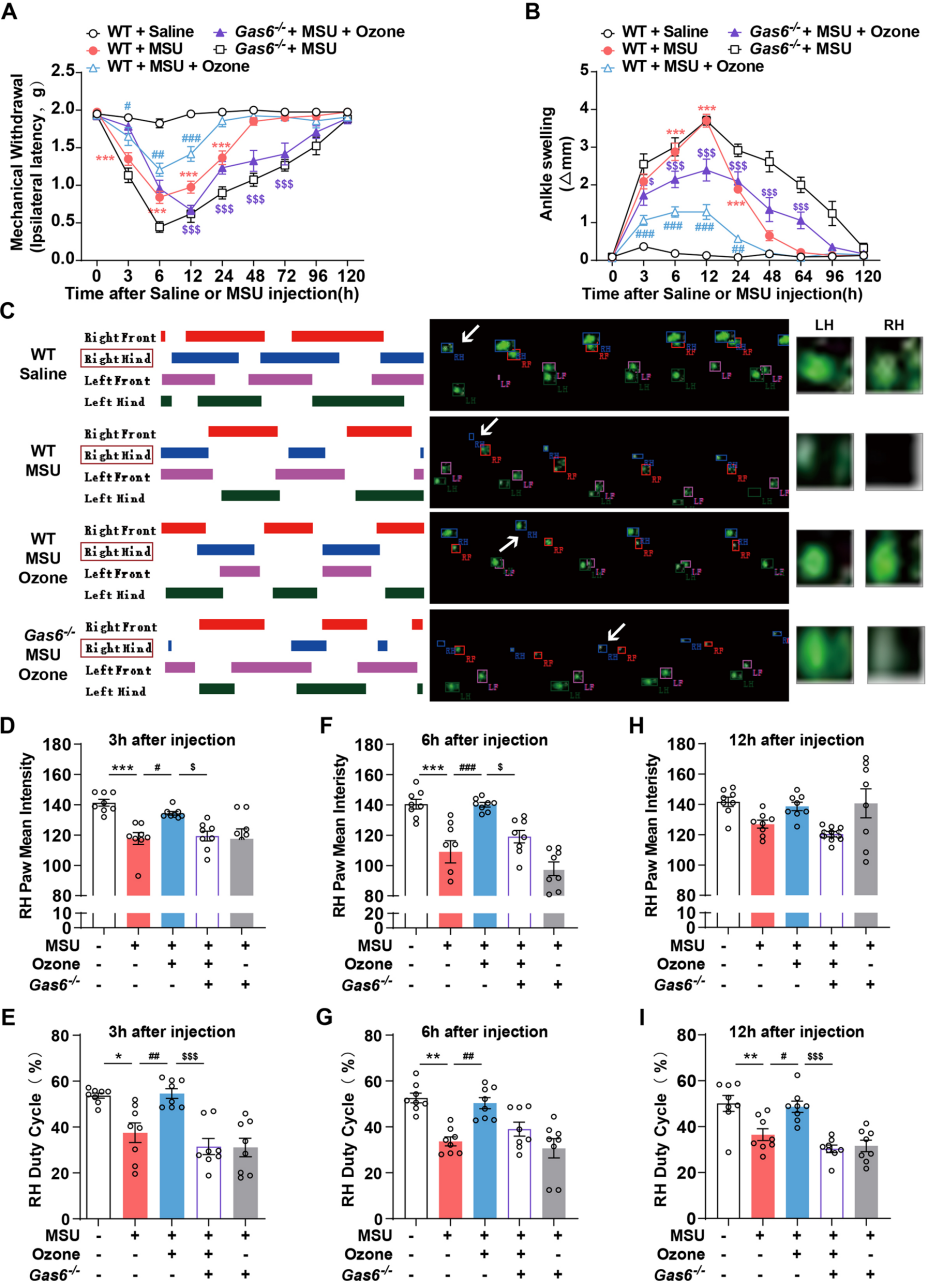
Gas6 played a key role in relieving gout pain with ozone. **A** Mechanical threshold of ipsilateral hind-paws between WT and Gas6-/- mice induced by MSU. **B** Ankle girth according to time after MSU crystal injection between WT and Gas6^−/−^ mice. **C**–**I** Ozone ameliorates the pain gait patterns of mice with MSU-treated. ^*^p < 0.05, ^**^p < 0.01 and ^***^p < 0.001 vs. control group; ^#^p < 0.05, ^##^p < 0.01 and ^###^p < 0.001 vs. MSU-treated group; ^&^p < 0.05, ^&&^p < 0.01 and ^&&&^p < 0.001 vs. MSU + ozone group

As an important ligand of MerTK, the previous data have confirmed that ozone plays an anti-inflammatory and analgesic role by activating AMPK-induced Gas6 expression and activating MerTK/SOCS3 signal pathway. So, we tried to better understand how important Gas6 to this pathway are. Gas6^−/−^ mice were used for subsequent experimental studies. Compared with the ozone treatment group, the analgesic effect of ozone was cancelled after Gas6 was knocked out. The withdrawal threshold of Gas6^−/−^ mice was significantly reduced at 6–72 h (Fig. 5A) and the swelling degree of the mouse ankle joint was significantly increased (Fig. 5B). The results of gait behavior showed that the therapeutic effect of ozone on the paw mean intensity and duty cycle was significantly cancelled after the elimination of Gas6. The above results show that Gas6 plays an important role in the process of ozone relieving gout.

### Decreased expression of serum Gas6 and protein S in patients

**Fig. 6:**
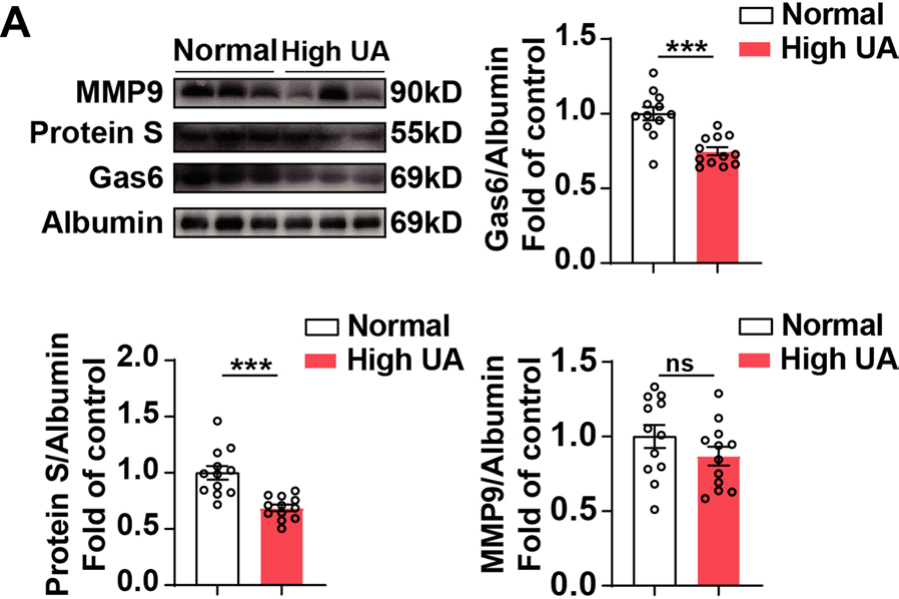
Decreased expression of plasma Gas6 and Protein S in patients. Gas6, Protein S and MMP9 levels in high UA patients and healthy controls. ^***^p < 0.001 vs. control group

Generally, a serum uric acid level of > 6.8 mg/dl is considered hyperuricemia [17], although not all patients with hyperuricemia will suffer from gout hyperuricemia is still necessary for the onset of gout [18]. Due to the limitation of available patient samples, we only selected the serum study of patients with hyperuricemia. Consistent with the blood sample data of gout mice, the expression level of Gas6 in the serum of patients was significantly decreased, which further confirmed the data of our animal experiment (Fig. 6).

## Discussion

Currently, a large number of studies have shown that pathogenic crystals can promote the transcription of NLRP3 inflammasome components [11, 19, 20]. MSU crystals are involved in inducing acute inflammation in gout through TLRs that stimulate the production of NLRP3 inflammasomes, interleukin-like factor and TNF-α. Meanwhile, many studies have demonstrated that inhibition of NLRP3 activation can effectively alleviate gouty arthritis, but its safety and efficacy need to be further verified by more clinical trials [21, 22]. However, ozone treatment for gout is a program that has been carried out in the pain departments of many hospitals in China, which effectively alleviate patients' pain.

Medical ozone as adjunctive therapy in the emergency of SARS-COV-2 infection has been proved by many clinicians [23]. Ozone therapy has been reported to reduce CRP, ESR, uric acid, and other biomarkers [24] and improve patients' VAS scores [25], indicating that it effectively reduces inflammation. Clinically, gouty arthritis is associated with severe inflammation, with symptoms such as joint pain and redness. Although clinical studies have shown that the use of ozone can effectively relieve severe pain caused by acute gout, its related mechanism has not been clarified. Our study showed that ozone inhibits TRLs signaling pathway by upregulating endogenous inflammatory brake SOCS3 to alleviate gout, which inhibit the activation of NLRP3 inflammasome at the upstream signaling pathway. We may provide a theoretical basis and academic foundation for the ozone treatment of gout pain in the clinic.

We observed that MSU crystals (100 μg/joint) can cause a strong inflammatory reaction in the ankle joint about 1 h after administration, which is manifested as ankle joint swelling and can induce mechanical hyperalgesia that lasts for 24 h. The pain reaction reached the maximum 6 h after the injection of MSU; the swelling reached the peak 12 h after the injection, and basically returned to normal 48 h after the injection (Fig. 1A, B). The clinical data confirmed this observation, because, in the human acute gout attack, severe pain and swelling developed rapidly, reaching a peak within 6–12 h. The intraperitoneal injection of 15 doses of ozone can reduce joint edema and harmful behavior. It is an important discovery that considering GA is a common inflammatory joint disease, pain during activity is the most common and unpleasant problem when gout attacks.

Impediment of walking is one of the main consequences of GA-related pain. Gait analysis for pain monitoring is increasingly used in humans. It has recently been tested in several rodent pain models, and gait abnormalities observed in human and rodent OA models show similar compensatory behavior. In this study, only 3 h after ozone treatment, we observed a significant reduction in paw mean intensity and duty cycle (Fig. 1D–F). Recent research results indicated that there are differences in the expression of receptors in skin sensory nerves compared with bones, and the withdrawal threshold obtained through von Frey filaments may not reflect the pain originating from bones and joints [26]. Although in the carrageenan (CAR) induced rat acute pain model, the von Frey results were highly correlated with the evaluation of single claw CatWalk parameters (paw mean intensity, foot print area, and duty cycle) [27]. We believe that the pain of GA mice may be worth evaluating along with gait changes.

Ozone significantly reversed the reduction of AMPK expression after ankle joint injection of MSU (Fig. 2E). Although the specific mechanism and signal pathway of ozone activating AMPK has not been fully clarified, some studies have shown that Ozone doses of 30 μg/ml and 40 μg/ml reduce ATP levels within 20 min [28]. It is known that ATP/AMP and ATP/ADP ratio are important factors affecting AMPK expression.

It must be emphasized that the beneficial therapeutic effect of ozone is based on the moderate energy regulation effect. The administration scheme and the treatment concentration of O_3_ are crucial to its therapeutic response. It can be seen from the fact that the therapeutic effect of an ozone dose of 30 μg/ml is less than that of 15 μg/ml (Fig. 1G–H, 2B–D). Ozone therapy can be very safe, when ozone is used within the therapeutic dose range, the reported complication has a rate of only 0.0007% and usually lasts for 30 min [29].

AMPK can inhibit oxidative stress and inflammation while mediating some of the therapeutic effects of metformin and the anti-inflammatory effects of methotrexate, salicylate, and high-dose aspirin [30]. Arhalofenate acid [31] and tanshinones [32] have also been reported to inhibit the activation of NLRP3 inflammatory bodies and the release of IL-1 induced via MSU by enhancing AMPK activity in macrophages. It has been reported that AMPK activators can inhibit experimental gout-like inflammation in vivo [7]. We further found that ozone's anti-inflammatory and analgesic effects can be partially canceled by the AMPK inhibitor CC in vivo and in vitro, blocking the Gas6/MerTK/SOCS3/MMP9 pathway (Figs. 2, 3).

The most well-known contribution of TAM receptors in dealing with inflammation is their significant role in efferocytosis. Effective efferocytosis may reprogram macrophages from an inflammatory state to an anti-inflammatory/Inflammation-diminishing state [33]. MerTK is the main mediator of efferocytosis. GAS6 can enhance the MerTK-dependent efferocytosis of human macrophages in vitro [34]. MerTK-deficient mice show ed exacerbated arthritis pathology [35]. During M2 polarization induced by dexamethasone, macrophages showed lower Axl protein level, while MerTK expression increased. Axl and MerTK may show different expression characteristics in an inflammatory environment [36]. Compared with Axl's inhibitor TP0903, the MerTK inhibitor UNC2541 is more obvious in eliminating the effect of ozone on reducing the increase of MMP9 induced by MSU in RAW264.7 (Fig. 3D, E). Therefore, we believe that Gas6 plays a protective role in acute gout symptoms by combining with MerTK. The plasma level of Gas6 in patients with high UA was significantly lower than that in the normal group (Fig. 6). The anti-inflammatory effect of Gas6 was further evaluated using Gas6 mice, and the knockout mice showed increased pain and inflammatory reaction (Fig. 6). These results showed that Gas6 was involved in the activation of MerTK, acting through internal negative feedback, and upregulating SOCS3 to inhibit MMP9.

Studies have shown that the activation of MMP2 and MMP9 in GA SF samples can reveal the inflammation of the knee joint in gouty arthritis [37]. In addition, SOCS3 plays a key role in regulating the chondrocyte response during inflammatory arthritis [38]. Considering that ozone can effectively alleviate osteoarthritis [39]. Therefore, we speculate and then confirm that up-regulation of SOCS3 and inhibition of inflammation are the molecular mechanism of ozone treatment of OA. The activation of the AMPK/Gas6/MerTK/SOCS3 signal pathway by ozone also provides new theoretical evidence for ozone treatment of osteoarthritis.

**Fig. 7:**
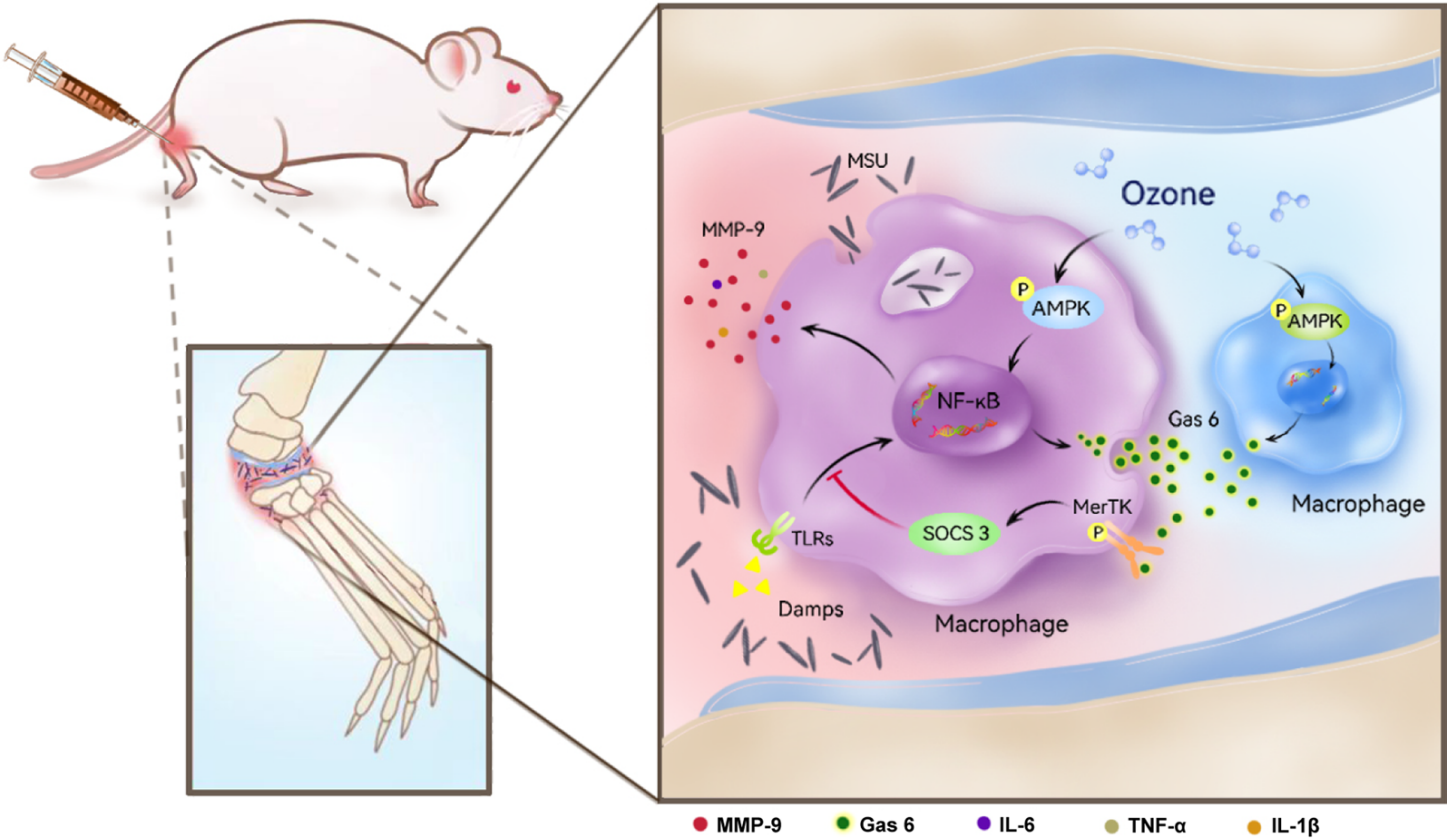
Schematic indicating that ozone reduces inflammation and alleviates gout pain by activating AMPK to up-regulate Gas6/MerTK/SOCS3 signaling pathway

In this study, we proposed the molecular mechanism of ozone in the treatment of gout pain through AMPK/Gas6/MerTK/SOCS3/MMP9 for the first time (Fig. 7). Gas6/MerTK/SOCS3 can prevent excessive and uncontrolled inflammatory responses to endogenous danger signals and provide new insights into the unique aspects of the human innate immune system. TAM can be a very promising target for managing GA inflammation and pain.

### Supplementary Information

**Additional file 1: Figure S1.** (A-C) Up/down-regulation of IL-1β, TNF-α and IL-6 mRNA in Raw264.7 cells were compared with the control group and MSU-treated group (n = 4). (D-E) Up/down secretion of IL-1β, IL-6 and TNF-α in Raw264.7 cells were compared with the control group and MSU-treated group. ^*^p < 0.05, ^**^p < 0.01 and ^***^p < 0.001 vs. control group; ^#^p < 0.05, ^##^p < 0.01 and ^###^p < 0.001 vs. MSU-treated group.

## Data Availability

The datasets during and/or analyzed during the current study are available from the corresponding author on reasonable request.

## Abbreviations

AGA: Acute gouty arthritis
AMPK: 5′-Adenosine monophosphate (AMP)-activated protein kinase
SOCS3: Suppressor of cytokine signaling 3
Gas6: Growth arrest specific protein 6
MerTK: Mer tyrosine kinase
MMP9: Matrix metallopeptidase 9
MSU: Monosodium urate
IL-1β: Interleukin 1β
TNF-α: Tumor necrosis factor α
IL-6: Interleukin 6
UA: Uricemia (UA)
