# Supplementary figures and images for: Ozone alleviates MSU-induced acute gout pain via upregulating AMPK/GAS6/MerTK/SOCS3 signaling pathway
Source: J Transl Med. 2023 Dec 8;21:890. doi: 10.1186/s12967-023-04769-1 (PMC10704676; doi:10.1186/s12967-023-04769-1)

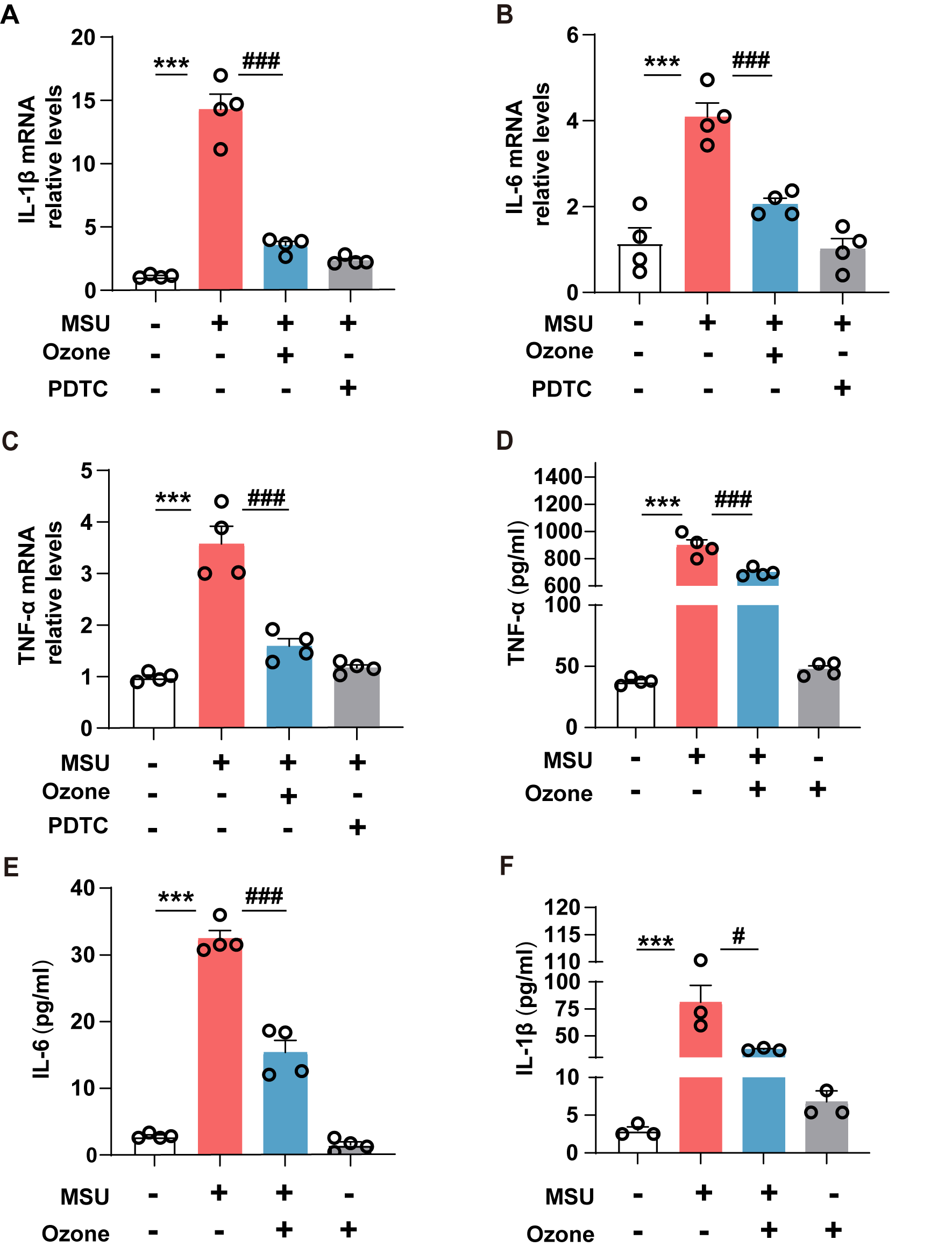

Supplement: Supplementary file 1 — Additional file 1: Figure S1. (A-C) Up/down-regulation of IL-1β, TNF-α and IL-6 mRNA in Raw264.7 cells were compared with the control group and MSU-treated group (n = 4). (D-E) Up/down secretion of IL-1β, IL-6 and TNF-α in Raw264.7 cells were compared with the control group and MSU-treated group. *p < 0.05, **p < 0.01 and ***p < 0.001 vs. control group; #p < 0.05, ##p < 0.01 and ###p < 0.001 vs. MSU-treated group. [file 12967_2023_4769_MOESM1_ESM.tif]
